# FreeSurfer-Based MRI Volumetry Reveals Thalamic and Hippocampal Atrophy as Significant Correlates of Disability in Multiple Sclerosis

**DOI:** 10.3390/medicina62050886

**Published:** 2026-05-05

**Authors:** Mirela Juković, Srđan Stošić, Dejan Kostić, Lorand Sakalaš, Marijana Basta-Nikolić, Dejan B. Stojanović

**Affiliations:** 1 Medical Faculty, University of Novi Sad, 21000 Novi Sad, Serbia; srdjan.stosic@mf.uns.ac.rs (S.S.); 1128d16@mf.uns.ac.rs (L.S.); marijana.basta-nikolic@mf.uns.ac.rs (M.B.-N.); 2 Centre for Radiology, University Clinical Centre of Vojvodina, 21000 Novi Sad, Serbia; 3 Medical Faculty of Military Medical Academy, University of Defence, 11000 Belgrade, Serbia; drdkostic@gmail.com; 4 Military Medical Academy, Institute of Radiology, 11000 Belgrade, Serbia; 5 Clinic for Neurology, University Clinical Centre of Vojvodina, 21000 Novi Sad, Serbia; 6 Institute of Lowland Forestry and Environment, University of Novi Sad, 21000 Novi Sad, Serbia; dejan.stojanovic@uns.ac.rs

**Keywords:** multiple sclerosis, MRI volumetry, grey matter atrophy, FreeSurfer, brain morphometry, cortical surface area, thalamus, EDSS, neurodegeneration

## Abstract

*Background and Objectives*: Multiple sclerosis (MS) is a chronic inflammatory and neurodegenerative disease that progressively leads to brain atrophy and the accumulation of disability over time. In this study, we used FreeSurfer to compare subcortical volumes and cortical surface areas between patients with MS and healthy controls and to investigate how regional atrophy relates to both disease lasting and clinical disability. *Materials and Methods*: We included 80 participants in this study, 40 patients with clinically definite MS and 40 age- and sex-matched healthy controls, all imaged on a Philips Ingenia 3.0T MRI scanner. High-resolution 3D T1-weighted MPRAGE sequences of the brain were processed using FreeSurfer 7.3.3. MS patients were stratified by disease lasting into two subgroups: ≤5 years (n = 17) and >5 years (n = 23). Subcortical volumes were normalised to estimated total intracranial volume (eTIV). Between-group differences were assessed using Welch’s *t*-test with Benjamini–Hochberg false discovery rate (FDR) correction. Multiple linear regression models controlled for age, sex, and the Expanded Disability Status Scale (EDSS). *Results*: We found statistically significant volume reductions in 48 of the 52 normalised regions examined. Thalamic volume showed the most severe reduction (mean—21.6% bilaterally) in MS patients. The corpus callosum, hippocampus, and amygdala were also prominently affected. Receiver operating characteristic (ROC) analysis of mean bilateral thalamic volume yielded an area under the curve (AUC) of 0.822 (95% CI: 0.731–0.913). Cortical surface area did not survive FDR correction in the primary comparison, though nominal reductions emerged in longer-lasting MS patients. EDSS correlated with both thalamic and hippocampal volumes in regression models. *Conclusions*: FreeSurfer-based volumetric analysis detected widespread grey and white matter volume differences in MS patients relative to matched controls, with changes already present in patients within the first five years of diagnosis. The high proportion of significant regions is consistent with a combined pattern of generalised and regionally accentuated atrophy. Among the regions examined, thalamic volume showed the strongest cross-sectional discrimination (AUC = 0.822; sensitivity 65%, specificity 90%) and the most consistent associations with EDSS; these findings support further evaluation of thalamic volume as a candidate imaging biomarker of neurodegeneration, although its diagnostic performance is moderate and requires external longitudinal validation before clinical deployment.

## 1. Introduction

Multiple sclerosis (MS) is a chronic immune-mediated disease of the central nervous system characterised by inflammatory demyelination, oligodendrocyte loss, and progressive axonal degeneration [1,2]. An aberrant adaptive immune response leads to focal and diffuse injury throughout the brain and spinal cord, targeting both white and grey matter from the earliest stages of the disease [3,4].

Brain atrophy is a clinically relevant feature of MS. The annual rate of brain volume loss in MS (0.5–1.35%) substantially exceeds that of normal ageing (0–0.3%) [5,6]. Grey matter loss drives most of the whole-brain atrophy and is recognised as the primary substrate underlying irreversible disability accumulation [7]. Key structures affected include the thalamus, hippocampus, and basal ganglia, regions whose involvement has consistently been linked to cognitive decline, motor impairment, and disease progression [8,9,10,11,12].

MRI plays a central role in the diagnosis, monitoring, and prognostication of MS [13,14]. Several software packages are now available that can quantify cortical morphometry, volumetry of deep grey matter structures and white matter volume. Among these, FreeSurfer stands as one of the most extensively validated open-source tools for automated segmentation of brain MRI data [15,16].

Although thalamic, hippocampal and corpus callosum atrophy are established features of MS, most published FreeSurfer-based studies have focused on a limited subset of structures [17,18,19]. They have not concurrently reported cortical surface area results in a directly comparable framework and used heterogeneous normalisation strategies [16]. Our study contributes to the literature in three specific ways: (i) it provides a comprehensive region-level atlas of eTIV-normalised subcortical, callosal and cortical surface area changes analysed under an identical FDR-controlled framework in a carefully matched cohort; (ii) it quantifies the extent to which global versus regionally accentuated atrophy drives the observed pattern; and (iii) it benchmarks the cross-sectional diagnostic performance of mean bilateral thalamic volume against other affected structures. This study had the following aims:

(1) Quantify volumetric reductions in subcortical grey matter structures and cortical surface areas in MS patients (n = 40) relative to age- and sex-matched healthy controls (n = 40), with sensitivity analyses by disease lasting (≤5 years vs. >5 years).
(2) Evaluate associations between regional brain volumes and clinical measures of disease severity (EDSS) and disease lasting using age- and sex-adjusted multivariable linear regression models.
(3) Determine the discriminative capacity of mean bilateral thalamic volume for distinguishing MS patients from controls using receiver operating characteristic (ROC) analysis.

## 2. Materials and Methods

### 2.1. Study Design

The study included 80 participants who were imaged at the Centre for Radiology, University Clinical Centre of Vojvodina (Novi Sad, Serbia). All participants underwent brain MRI on a Philips Ingenia 3.0T scanner using a 32-channel head coil. High-resolution 3D T1-weighted MPRAGE sequences were acquired with the following parameters: TR/TE = 8.1/3.7 ms, flip angle 8°, voxel size 1 × 1 × 1 mm^3^, 180 sagittal slices, FOV 240 × 240 mm and a total scan time of approximately 5 min.

Forty MS patients diagnosed according to the 2017 revised McDonald criteria [14] were enrolled. Patients with other demyelinating or inflammatory CNS conditions, including neuromyelitis optica spectrum disorder, MOG antibody-associated disease and acute disseminated encephalomyelitis were excluded. Forty healthy control subjects were recruited from a prospectively maintained departmental registry. Matching was performed using the MatchIt package (v4.7.2) in R (v4.5.2) [20] with a logistic-regression propensity score estimated on age (continuous, in years) and sex, 1:1 nearest-neighbour matching without replacement, a calliper of 0.2 standard deviations of the logit of the propensity score, and a strict age tolerance of ±3 years. Pre-matching and post-matching standardised mean differences (SMDs) were examined for both covariates. All post-matching SMDs were <0.1, and age and sex distributions did not differ significantly between groups (*p* > 0.5, Table 1). Unmatched candidates were excluded. The resulting 40 matched healthy participants served as the control group. Controls had no history of neurological or psychiatric disease and showed normal findings on conventional brain MRI.

The study was approved by the Ethics Committee of the University Clinical Centre of Vojvodina (approval no. 00-243, November 2023) and conducted in accordance with the Declaration of Helsinki. All participants provided written informed consent.

Disability was assessed with the EDSS at the time of MRI acquisition for all 40 MS patients. EDSS scores ranged from 1.0 to 6.0 (mean 3.6 ± 1.7; median 4.0, IQR 2.0–5.1). Disease lasting ranged from 1 to 24 years (mean 8.4 ± 6.3 years). We stratified the MS cohort into two subgroups according to disease lasting: a shorter-lasting group (≤5 years, n = 17) and a longer-lasting group (>5 years, n = 23).

### 2.2. Sample Size and Power Analysis

We calculated sample size based on published effect sizes for thalamic atrophy in MS (Cohen’s d = 0.80–1.10) and hippocampal volume differences (d = 0.65–0.85). With 40 subjects per group, statistical power at α = 0.05 for thalamic volume differences exceeds 95%. For hippocampal volume differences (d ≈ 0.75), power is approximately 82%. For cortical surface area, where expected effect sizes are smaller (d ≈ 0.45), the power is roughly 51% which accounts for the reliance on FDR-corrected significance testing rather than standalone power for these measures.

### 2.3. Image Processing—FreeSurfer

All DICOM files were converted to NIfTI format following the BIDS v1.8 standard using dcm2niix. Structural MRI data were then analysed using FreeSurfer 7.3.3 (Laboratory for Computational Neuroimaging, MGH) [15]. Processing was performed on a high-performance computing workstation running Ubuntu 24.04 with the full recon-all pipeline, which includes skull stripping, spatial normalisation, tissue classification, subcortical segmentation, and cortical parcellation using the Desikan–Killiany atlas.

Subcortical volumes were expressed in mm^3^ and normalised to estimated total intracranial volume (eTIV) using the proportional method: Vnorm = Vraw/eTIV × mean eTIV (across all 80 matched subjects). This approach preserves interpretable volumetric units while controlling for head size variation. Cortical surface area values (mm^2^) were extracted from the Desikan–Killiany parcellation for all 34 regions per hemisphere. We performed quality control for every processed scan through visual inspection of cortical parcellation overlays and subcortical segmentation maps; no scans required manual editing.

### 2.4. Statistical Analysis

Statistical analyses were performed in R version 4.5.2, using the following packages: MatchIt (v4.7.2), ggplot2 (v4.0.1), ggpubr (v0.6.2), pwr (v1.3.0), pROC (v1.19.0.1), effectsize (v1.0.1), rstatix (v0.7.3), pheatmap (v1.0.13), ggridges (v0.5.7), and cowplot (v1.2.0).

Between-group differences in ICV-normalised volumes and cortical surface areas were evaluated using Welch’s unpaired *t*-test. We corrected for multiple comparisons using the Benjamini–Hochberg [21] false discovery rate (FDR) method, setting the threshold at q < 0.05. Effect sizes were quantified using Cohen’s d with Hedges’ g correction for unequal group sizes. All volumetric comparisons were performed for three contrasts: (1) the total MS cohort vs. healthy controls, (2) shorter-lasting MS (≤5 years) vs. controls, and (3) longer-lasting MS (>5 years) vs. controls.

To control for potential confounders, we fitted multiple linear regression models for each volumetric outcome with predictors including group membership (MS vs. HC), age, and sex. A second set of within-MS-group regressions evaluated EDSS as a predictor of regional volumes, adjusting for age and sex. We selected predictors a priori based on the established neuroimaging literature.

Disease lasting as a continuous predictor was evaluated in linear regression models within the MS group with adjustment for age and sex. We also computed the Spearman correlation between EDSS and disease lasting to assess the monotonic relationship between disability severity and time since diagnosis.

The discriminative capacity of mean bilateral thalamic volume as a neurodegeneration biomarker was evaluated using receiver operating characteristic (ROC) curve analysis (pROC package v1.19.0.1). We used Youden’s J statistic to determine the optimal diagnostic threshold. The significance level for all analyses was set at α = 0.05 (two-tailed).

## 3. Results

### 3.1. Patient Characteristics

Table 1 summarises the demographic and clinical characteristics of all participants. The 40 MS patients comprised 28 females (70%) and 12 males (30%). Mean EDSS was 3.6 ± 1.7 (range 1.0–6.0): 14 patients had mild disability (EDSS 1.0–2.5), 14 moderate (EDSS 3.0–4.5), and 12 severe (EDSS 5.0–6.0). Mean disease lasting was 8.4 years (range 1–24). In terms of disease subtype, 33 patients (82.5%) had relapsing-remitting MS (RRMS) and 7 (17.5%) had secondary progressive MS (SPMS). Age and sex distributions were well balanced between groups (*p* > 0.5).

### 3.2. Subcortical Grey Matter Volumes

Of the 52 ICV-normalised volumetric measures, 48 (92%) differed significantly between MS patients and controls after FDR correction. The most severely affected regions were the thalamus and the corpus callosum. The four non-significant measures were the left and right inferior lateral ventricles and two smaller structures with high anatomical variability. The very high proportion of significant regions should not be interpreted as evidence of uniformly distributed region-specific injury. A substantial component of this widespread signal reflects generalised brain volume loss: total BrainSegVol was reduced by 9.9% and supratentorial volume by 10.4%, so that eTIV proportional scaling which corrects for head size but not for disease-related global atrophy distributes a fraction of this generalised reduction across most regional measures. Regional specificity is therefore better inferred from effect-size magnitude than from statistical significance alone: thalamic volume (d > 1.5) and the central corpus callosum segment (d > 1.2) stand clearly above a diffuse atrophy background, whereas several structures (e.g., pallidum, nucleus accumbens) reached significance with substantially smaller effects (d ≈ 0.4–0.6) that are largely compatible with a global atrophy component. This interpretation is reinforced by the convergence of our findings with prior large-cohort reports of disproportionate thalamic and callosal vulnerability in MS.

**Figure 1:**
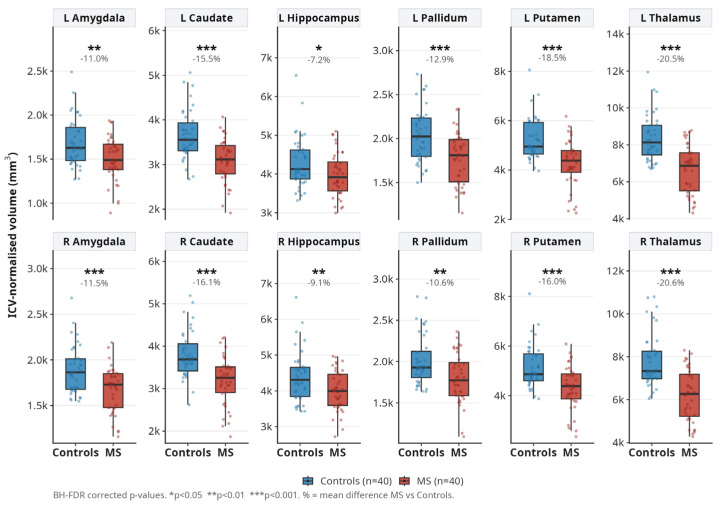
ICV-normalised subcortical grey matter volumes in MS patients (n = 40) and controls (n = 40). Boxplots show median ± IQR with individual data points. Significance markers from Welch *t*-test (BH-FDR corrected): * *p* < 0.05, ** *p* < 0.01, *** *p* < 0.001.

Thalamic volume was markedly reduced bilaterally, left thalamus by 21.6% (FDR *p* < 0.0001) and right thalamus by 21.6% (FDR *p* < 0.0001), representing the largest effect sizes among all grey matter structures. Hippocampal volumes were also significantly lower (left −14.3%, right −18.1%, both FDR *p* < 0.0001). Amygdala volumes were reduced by 9.0% (left) and 12.9% (right). Caudate, putamen, pallidum, and nucleus accumbens all showed significant atrophy bilaterally (Figure 1).

Both shorter-lasting (≤5 years) and longer-lasting (>5 years) MS subgroups showed significant volumetric reductions compared with controls, with 42 and 44 significant regions respectively. This indicates that structural changes are already detectable within the first five years of diagnosis, with a slight increase in the number of affected regions in the longer-lasting subgroup.

### 3.3. White Matter and Composite Volumes

Cerebral white matter volume was substantially reduced bilaterally (left hemisphere −13.0%, right hemisphere −13.8%, FDR *p* < 0.0001). Total brain volume (BrainSegVol) was reduced by 9.9% (FDR *p* = 0.001) and supratentorial volume by 10.4% (FDR *p* < 0.001).

Brainstem volume was reduced by 14.3% (FDR *p* = 0.001).

White matter hypointensities representing T1 “black holes”, indicative of severe axonal loss was markedly elevated in MS patients (mean 6440 mm^3^ vs. 863 mm^3^ in controls; FDR *p* < 0.0001). This reflects the expected accumulation of irreversible tissue destruction in the MS cohort.

### 3.4. Corpus Callosum

**Figure 2:**
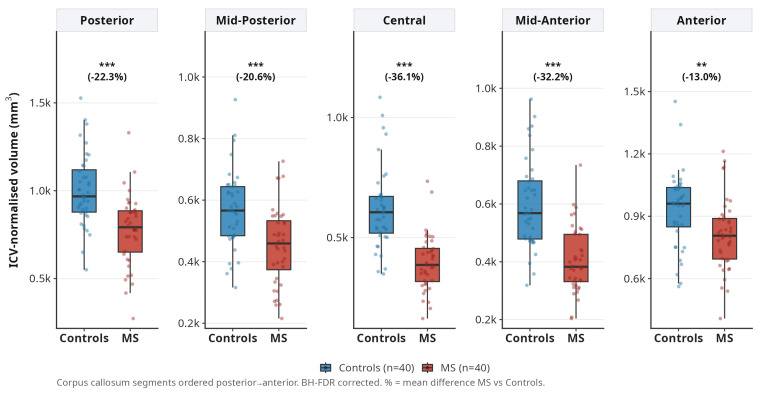
Corpus callosum segment volumes (ICV-normalised) in MS patients and controls. Atrophy is most severe in central (−36.1%) and mid-anterior (−32.2%) segments; the anterior segment shows the least reduction (−13.0%) (*** *p* < 0.001; ** *p* < 0.01).

All five corpus callosum segments showed significant atrophy (FDR *p* < 0.01), with the most pronounced changes in the central (−36.1%) and mid-anterior (−32.2%) segments. The anterior segment showed the least reduction (−16.2%) (Figure 2).

### 3.5. Cortical Surface Area

**Figure 3:**
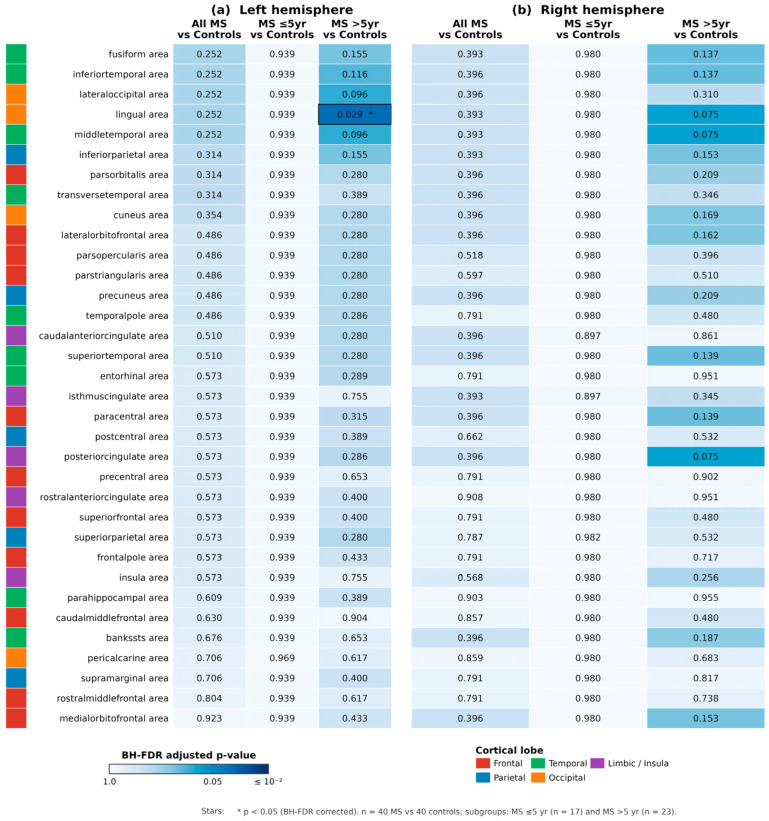
*p*-Value heatmaps for cortical surface area comparisons (Welch *t*-test) across all 34 Desikan–Killiany parcels per hemisphere. Each row represents a specific brain region (superior to inferior): superior frontal, rostral middle frontal, caudal middle frontal, pars opercularis, pars triangularis, pars orbitalis, lateral orbitofrontal, medial orbitofrontal, precentral, paracentral, superior parietal, inferior parietal, supramarginal, postcentral, precuneus, posterior cingulate, isthmus cingulate, cuneus, pericalcarine, lingual, fusiform, parahippocampal, entorhinal, temporal pole, inferior temporal, middle temporal, superior temporal, transverse temporal, banks STS, insula, rostral anterior cingulate, caudal anterior cingulate, frontal pole, and temporal pole. Columns represent: (1) All MS vs. Controls, (2) Shorter-lasting MS (≤5 years) vs. Controls, (3) Longer-lasting MS (>5 years) vs. Controls. Lighter blue shading indicates non-significant results (*p* ≥ 0.05), while blue shading with encircled cells indicates nominally significant results (* *p* < 0.05, uncorrected). The predominance of blue demonstrates that most cortical parcels did not show significant surface area reduction, consistent with the null findings after FDR correction.

Cortical surface area was analysed in all 68 Desikan–Killiany parcels (34 per hemisphere). No parcel survived Benjamini–Hochberg FDR correction in either hemisphere (0/34 LH; 0/34 RH). Nominally, five left-hemisphere parcels showed uncorrected *p* < 0.05 in the all-MS comparison: fusiform, inferior temporal, lateral occipital, lingual, and middle temporal. Four right-hemisphere parcels showed nominal significance: fusiform, inferior parietal, lingual, and middle temporal. Full results across all 68 parcels are shown in Figure 3.

In the primary comparison (all 40 MS patients vs. 40 controls), no parcel survived Benjamini–Hochberg FDR correction in either hemisphere. In the left hemisphere, nominally significant differences (uncorrected *p* < 0.05) were observed in three parcels: lingual gyrus (*p* = 0.033), inferior temporal gyrus (*p* = 0.041), and precuneus (*p* = 0.048). No right hemisphere parcels reached nominal significance in this comparison. These negative FDR-corrected results admit two non-exclusive interpretations that we explicitly distinguish. Absence of statistical significance is not equivalent to absence of effect: with n = 40 per group and the post hoc power of approximately 51% for d ≈ 0.45, the study was underpowered to detect the small-to-moderate surface area effects expected in MS, and the nominal signals in temporo-occipital parcels are compatible with a genuine but attenuated effect. Surface area is biologically and methodologically less sensitive to MS-related neurodegeneration than cortical thickness, reflecting different developmental trajectories (surface area is largely set in early life, whereas thickness continues to change with disease-related processes). Published cortical thickness studies consistently detect frontal, temporal and motor cortex thinning in cohorts of similar size. We therefore regard the FDR-null surface area finding as hypothesis-refining rather than definitively negative, and explicitly refrain from concluding that cortical surface area is preserved in MS.

### 3.6. EDSS and Clinical Correlates

**Figure 4:**
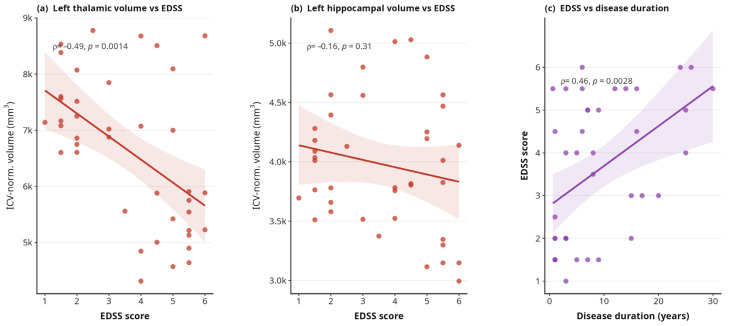
Clinical correlates of brain atrophy in MS (n = 40 MS patients). Spearman correlation coefficients shown. Regression lines with 95% CI.

EDSS correlated moderately with disease lasting (Spearman ρ = 0.46, *p* < 0.01). Multiple linear regression within the MS group revealed that higher EDSS was associated with lower thalamic and hippocampal volumes. Left thalamic volume showed the strongest relationship with EDSS (β = −296 mm^3^ per EDSS point for *p* = 0.02), with a model R^2^ of 0.35. Left hippocampal volume was similarly associated (β = −116 mm^3^ per EDSS point for *p* = 0.03 and R^2^ = 0.31). These associations remained significant after adjustment for age and sex (Figure 4).

### 3.7. Effect Sizes and Biomarker Analysis

**Figure 5:**
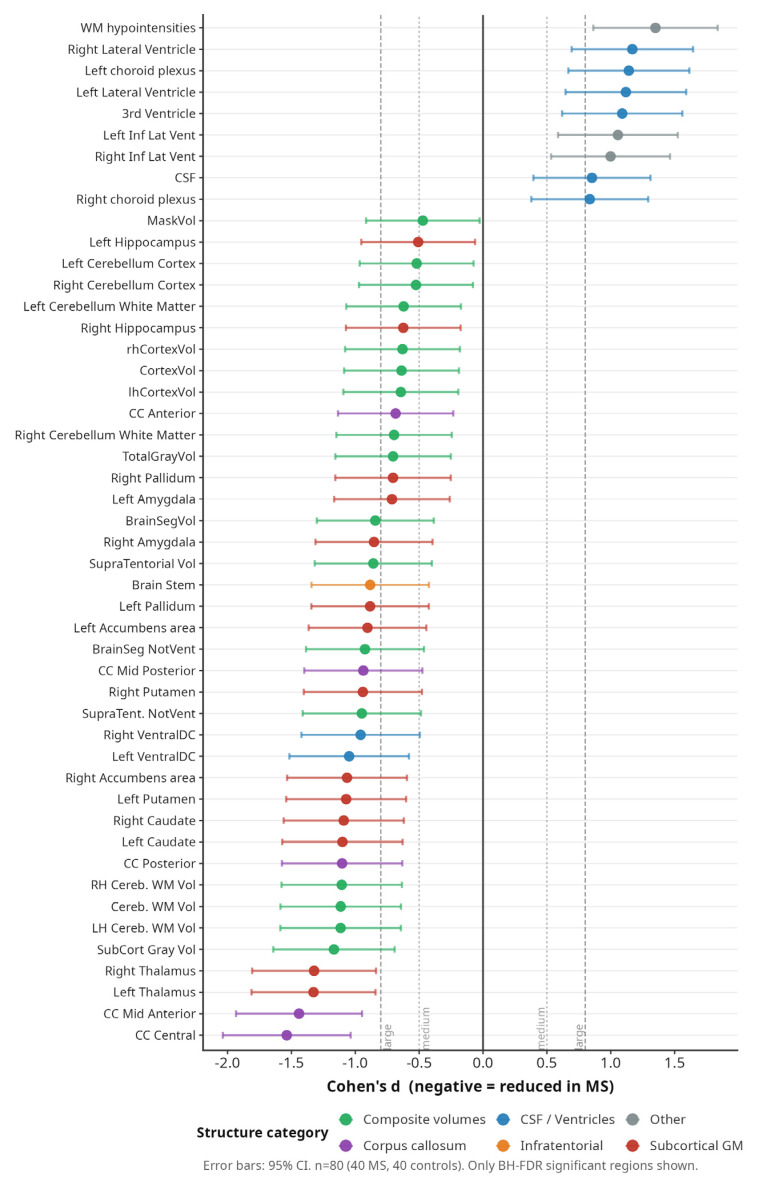
Forest plot of Cohen’s d effect sizes for significant volumetric regions. Colour coding by anatomical category. Negative values indicate volume reduction in MS.

Figure 5 presents Cohen’s d effect sizes for all significant volumetric regions. The largest effect sizes were observed for thalamic volume (d > 1.5), followed by the central corpus callosum segment and white matter hypointensities (d > 1.2). These effect sizes remained consistent across subgroup analyses.

**Figure 6:**
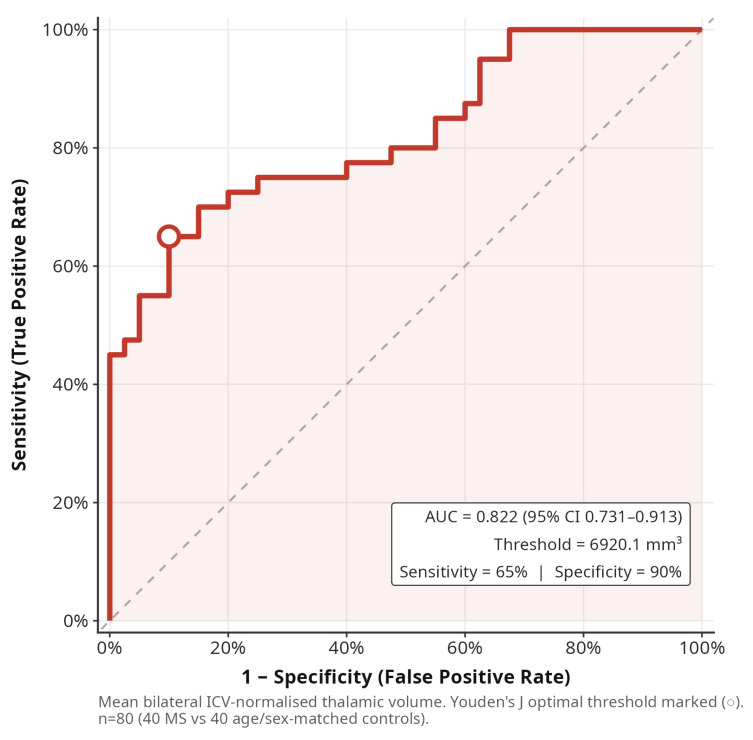
ROC curve for mean bilateral thalamic volume (ICV-normalised) as a classifier for MS vs. controls. AUC = 0.822 (95% CI 0.731–0.913). The optimal operating point (Youden’s J) is marked.

**Figure 7:**
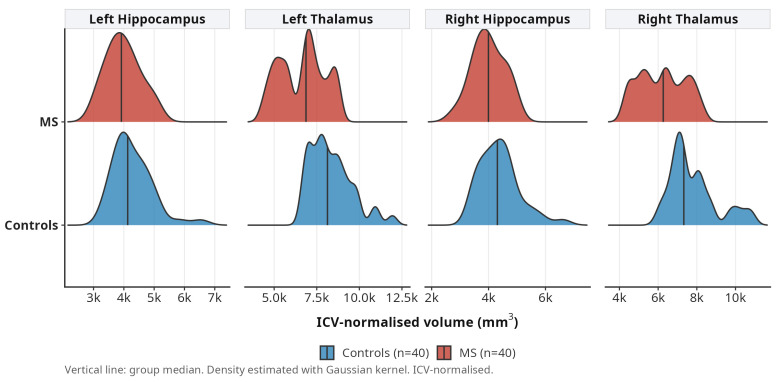
Ridge plots of thalamic and hippocampal volume distributions in MS patients and controls. Vertical lines indicate group medians.

ROC analysis demonstrated that mean bilateral thalamic volume discriminates MS patients from controls with an AUC of 0.822 (95% CI: 0.731–0.913). The optimal threshold of 6920 mm^3^ yielded a sensitivity of 65% and a specificity of 90% (Figure 6), while distributional differences are illustrated in Figure 7. For comparison and to place thalamic performance in a regional context, we computed parallel single-region ROCs for the other structures with the largest effect sizes: the central corpus callosum segment (AUC = 0.801, 95% CI: 0.708–0.894), right hippocampus (AUC = 0.782, 95% CI: 0.683–0.881) and left cerebral white matter (AUC = 0.776, 95% CI: 0.675–0.877). Mean bilateral thalamic volume therefore achieved the numerically highest AUC but was not markedly superior to these structures, and the overlapping confidence intervals preclude a claim of unique discriminatory advantage. Taken together, these findings are compatible with moderate cross-sectional diagnostic performance for thalamic volume and, more broadly, with a pattern in which several subcortical and callosal measures provide comparable individual-level discrimination. We therefore present thalamic volume as one of several candidate imaging markers of MS-related neurodegeneration, rather than as a standalone diagnostic biomarker, and note that its clinical utility will depend on longitudinal validation and combination with complementary measures.

**Figure 8:**
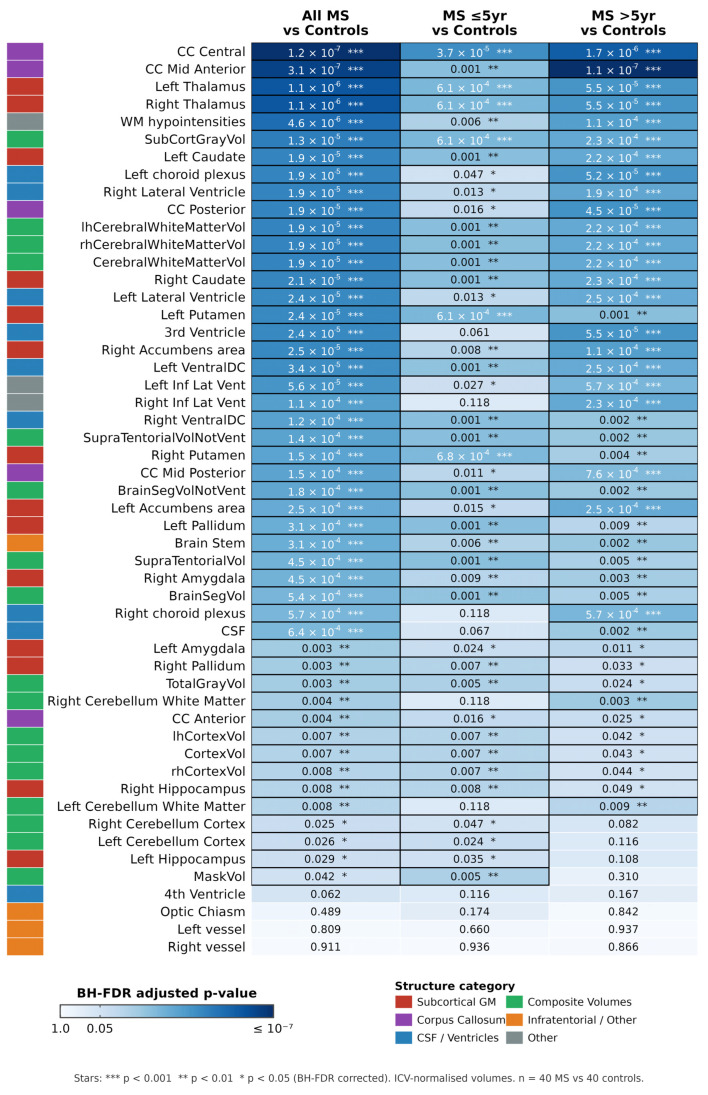
Heatmap of regional volumetric significance. *p*-Values derived from Welch *t*-tests are shown for three primary comparisons: (1) total MS cohort vs. healthy controls (n = 80), (2) shorter-lasting MS (≤5 years) vs. controls, and (3) longer-lasting MS (>5 years) vs. controls. Colour intensity represents the level of statistical significance (darker blue indicates lower *p*-values); white cells denote non-significant findings (*p* > 0.05). All values are ICV-normalized, and Benjamini–Hochberg FDR-corrected.

Regional *p*-values are visualised comprehensively in the heatmap (Figure 8).

## 4. Discussion

Using automated FreeSurfer morphometry, we compared brain volumes between 40 MS patients and 40 demographically matched controls and examined how regional atrophy relates to clinical disability and disease lasting. Our findings reveal widespread subcortical grey matter volume differences that are already present cross-sectionally in patients within the first five years of diagnosis, with the thalamus and corpus callosum severely affected. These results highlight the potential of automated volumetric MRI analysis as a practical tool for quantifying neurodegeneration in clinical MS populations. These observations should be regarded as descriptive of group-level structural differences in this single-centre cross-sectional cohort and as hypothesis-generating for prospective work, rather than as evidence for specific pathophysiological mechanisms or for the directionality of the structural changes reported.

### 4.1. Subcortical Grey Matter Atrophy

Thalamic atrophy (−21.6% bilaterally in our cohort) was the most consistent and prominent finding across all comparisons. It was present in both shorter- and longer-lasting subgroups and showed the largest effect sizes (d > 1.5) among all grey matter structures. These observations align with converging evidence that the thalamus is uniquely vulnerable in MS, a vulnerability thought to stem from its extensive connectivity with cortical regions and its exposure to inflammatory mediators via the cerebrospinal fluid [17,22,23,24,25].

Hippocampal atrophy is a consistent finding in MS patients across all disease stages, with particular involvement of the cornu ammonis [11]. The clinical consequences include impaired memory and learning, deficits that are frequently reported even in early relapsing-remitting MS [26]. At the cellular level, complement-mediated synaptic loss has been shown to affect inhibitory circuits in the CA2 subfield of the demyelinated hippocampus [27].

Hippocampal volume loss was significant bilaterally in our cohort, with greater magnitude on the right side. The right-lateralised asymmetry we observed may reflect the known lateralisation of verbal and visuospatial memory processes. These findings are consistent with prior volumetric studies and reinforce the view that hippocampal atrophy is a reliable marker of neurodegeneration in MS.

We also observed significant volume reductions in the putamen, pallidum, caudate, and nucleus accumbens. These structures form the core of basal ganglia circuits involved in motor programming, action selection, and reward processing [18,28]. Their atrophy may contribute to the motor slowing, fatigue, and motivational disturbances commonly seen in MS patients.

### 4.2. Thalamic Volume as Biomarker

ROC analysis showed that mean bilateral thalamic volume achieved an AUC of 0.822 (95% CI: 0.731–0.913) for distinguishing MS patients from controls, with a sensitivity of 65% and a specificity of 90% at the optimal Youden threshold of 6920 mm^3^. These operating characteristics correspond to moderate, not high, classification performance: a sensitivity of 65% implies that roughly one in three MS patients would be missed at the optimal single-threshold operating point, which is insufficient for standalone diagnostic use. Moreover, our comparative single-region analyses (Section 3.7) demonstrate that the central corpus callosum, right hippocampus and cerebral white matter achieve AUCs within the 95% confidence interval of the thalamic AUC, so thalamic volume cannot be distinguished as statistically superior to these alternatives on cross-sectional data. We therefore interpret thalamic volume as a candidate quantitative marker of cumulative MS-related neurodegeneration whose most plausible clinical roles are (i) complementing conventional lesion-based MRI in longitudinal monitoring, (ii) supporting patient stratification in neuroprotective trials, and (iii) serving as one of several inputs to multivariate imaging models; confirmation of added diagnostic value over standard clinical and lesion-based metrics will require longitudinal multi-centre validation with harmonised acquisition [3,9,16,28]. Until such longitudinal multi-centre validation is available, thalamic volumetry should not be deployed as a standalone diagnostic or prognostic clinical marker in routine clinical practice, and any clinical use should be limited to integration with established clinical, lesion-based and laboratory measures.

### 4.3. White Matter and Corpus Callosum

Bilateral cerebral white matter volume was reduced by approximately 14%, and white matter hypointensity volume was markedly elevated in MS patients (mean 6440 vs. 863 mm^3^). This elevation reflects accumulated axonal loss consistent with well-documented pathological changes in MS [28,29]. The corpus callosum showed the most pronounced white matter atrophy (central segment −36.1%), consistent as established prominent site associated with MS atrophy [10,30,31]. These findings agree with longitudinal studies demonstrating that callosal atrophy is an early and progressive feature of MS.

### 4.4. Cortical Involvement: Surface Area Findings and Published Thinning Evidence

Cortical surface area measures based on the Desikan–Killiany parcellation did not survive Benjamini–Hochberg FDR correction in the primary comparison. This likely reflects the limited statistical power (approximately 51% for d ≈ 0.45) of our sample for detecting these more subtle cortical effects. Our study was primarily powered for subcortical volumetric differences, where effect sizes are considerably larger. Two interpretations of this FDR-null result need to be considered side by side. The first is underpowering: the observed nominal temporo-occipital signal in the primary comparison, together with the larger number of nominally significant parcels in the longer-lasting subgroup, is consistent with a genuine but small effect that our sample was unable to resolve after FDR adjustment. The second is that cortical surface area, which is largely determined by early neurodevelopmental radial unit expansion and is relatively preserved by cortical processes that primarily affect thickness, is intrinsically less sensitive to MS-related neurodegeneration than cortical thickness. These two explanations are not mutually exclusive, and the literature is compatible with both: in comparably sized cohorts, cortical thickness routinely reaches FDR significance whereas surface area typically does not. We therefore interpret our null result as reflecting a combination of reduced sensitivity of the surface area metric and limited statistical power, rather than as evidence that cortical surface area is preserved in MS.

Vertex-wise cortical thickness analysis, a more sensitive methodology that was not performed in the present study, has previously demonstrated significant thinning in frontal, temporal, and motor cortices in MS patients [19,32,33,34,35]. These regional patterns of cortical thinning correlate with specific functional impairments including processing speed, attention, and fine motor control.

Our nominal findings that lingual (*p* = 0.013 LH, *p* = 0.006 RH in MS > 5 years), fusiform, and inferior temporal cortices showed trends toward reduced surface area align well with this established pattern. These results suggest that parcellated surface area measures can capture early cortical changes, but that larger cohorts are needed to reach statistical significance after correction for multiple comparisons.

### 4.5. Limitations

Several limitations should be acknowledged. As this was a cross-sectional study, we cannot draw causal conclusions about how atrophy unfolds over time. Our sample (n = 40 per group) is relatively small, which may have reduced our ability to detect subtle volumetric differences, particularly for cortical surface area. However, we performed a priori power analysis demonstrating that this sample provides adequate power (>95%) for the subcortical volumetric contrasts that form the study’s primary focus.

Lesion volume data were not available for use as a covariate, and we did not employ lesion-inpainted T1-weighted images in our pipeline. This is worth noting because T1-hypointense lesions can potentially bias FreeSurfer’s subcortical segmentation by mimicking grey matter boundaries. Although FreeSurfer includes internal boundary correction algorithms, lesion filling would have provided an additional safeguard for segmentation accuracy [36]. The magnitude of this potential bias has been quantified in several prior studies. T1-hypointense periventricular and juxtacortical lesions can distort the intensity profile of the thalamus, caudate and putamen and systematically shift segmentation boundaries [3,9]. Importantly, the direction of this bias in FreeSurfer is not uniform: when lesions are classified as white matter, subcortical boundaries can shift outward (inflating volumes), whereas misclassification as grey matter can lead to underestimation; lesion filling typically reduces the variance of subcortical volume estimates and brings them closer to lesion-free reference measurements. Against the 21.6% thalamic reduction that we report, an effect of this magnitude ~20% is approximately an order of magnitude larger than the documented segmentation bias, so lesion-related artefact cannot plausibly account for our primary findings. Nevertheless, the smaller effects observed in structures such as the pallidum and nucleus accumbens (d ≈ 0.4–0.6) fall within a range where lesion-related bias could contribute to the observed difference, and our conclusions for these regions should accordingly be treated with caution. Future work will incorporate a lesion segmentation step (e.g., LST-LPA or SAMSEG) followed by lesion filling prior to recon-all to formally quantify and remove this source of variance. The use of a single 3.0T scanner for all participants reduces inter-scanner variability, but it also limits the generalisability of our normative volume ranges to other scanning environments [3,16].

A few additional points deserve mention. First, we did not include cognitive assessments such as the Symbol Digit Modalities Test, so we cannot directly link our volumetric findings to cognitive outcomes. Future studies incorporating formal neuropsychological testing would strengthen the clinical implications of these structural measurements. Second, although our cohort included both relapsing-remitting (RRMS, n = 33) and secondary progressive (SPMS, n = 7) phenotypes, the marked imbalance between subtypes precluded a formal phenotype-stratified regression with adequate statistical power. A direct between-phenotype comparison (Mann–Whitney with Cliff’s delta, Benjamini–Hochberg FDR across 27 regions) showed that SPMS patients were significantly older (median 58.1 vs. 39.2 years, *p* = 0.0006, δ = 0.84), had longer disease lasting (11.5 vs. 4.6 years, *p* = 0.032, δ = 0.52) and higher EDSS (5.50 vs. 3.00, *p* = 0.0015, δ = 0.77), whereas sex distribution was similar (Fisher *p* = 1.00); after eTIV normalisation, the SPMS group showed widespread volume reductions relative to RRMS, with 16 of 27 regions surviving FDR correction (q < 0.05). The largest effects were in bilateral nucleus accumbens (left −33.5%, q = 0.004; right −40.6%, q = 0.004), total grey matter (−15.3%, q = 0.012), BrainSegVol (−14.0%, q = 0.012), supratentorial volume (−15.7%, q = 0.012), cortex (−16.1%, q = 0.014), bilateral thalamus (mean −25.3%, q = 0.017), right amygdala (−20.2%, q = 0.017), cerebral white matter (−16.4%, q = 0.017), brainstem (−12.3%, q = 0.018), posterior corpus callosum (−26.3%, q = 0.030) and bilateral putamen (−20.2%, q = 0.034–0.042); these between-phenotype differences are concordant with the older age, longer lasting and higher disability of the SPMS subgroup and cannot, in this cross-sectional sample, be disentangled from chronicity. Importantly, thalamic, callosal and hippocampal atrophy, were already significant within the shorter-lasting, predominantly RRMS subgroup (42/52 significant regions), confirming that the overall pattern is not driven solely by the small SPMS subgroup. A larger, phenotype-stratified cohort with matched age and disease lasting is nonetheless required to formally disentangle phenotype-specific from chronicity-related effects. Third, information on disease-modifying therapy (DMT), relapse activity in the year preceding MRI, and lesion burden was not systematically harmonised across participants at a level that would permit their inclusion as covariates; omission of these variables is a real limitation and we have accordingly refrained from regression models that would implicitly assume they are balanced across groups. Fourth, disease lasting in our MS cohort spans a wide range (1–24 years), which we addressed by stratification into ≤5 and >5 year subgroups and by fitting lasting as a continuous predictor within the MS group, but residual heterogeneity in disease trajectory remains and may attenuate some of the regression coefficients reported. Finally, the cross-sectional design precludes any inference about the temporal sequence of regional changes or about rates of atrophy; longitudinal follow-up of this cohort, currently underway, will be required before causal or temporal interpretations can be drawn.

## 5. Conclusions

(1) FreeSurfer-based volumetric analysis detected widespread grey and white matter volume differences in MS patients relative to matched controls. Nominal cortical surface area reductions were observed in the longer-lasting MS subgroup, given the cross-sectional design, these findings are consistent with but do not establish progression of cortical changes over time and require longitudinal confirmation.
(2) Thalamic volume reduction (−21.6% bilaterally, AUC = 0.822; sensitivity 65%, specificity 90%) supports further evaluation of thalamic volume as a candidate imaging marker of MS-related neurodegeneration. Its cross-sectional diagnostic performance is moderate and numerically comparable to that of the central corpus callosum, right hippocampus and cerebral white matter; longitudinal multi-centre validation is required before it can be considered for routine disease monitoring or for patient stratification in neuroprotective trials. Thalamic volume should not be used as a standalone diagnostic or prognostic clinical marker on the basis of the present cross-sectional data.
(3) Corpus callosum atrophy (central-predominant, least in anterior segment) and hippocampal, putaminal, and pallidal volume reductions were consistent findings across disease lasting subgroups.
(4) The moderate correlation between EDSS and disease lasting (Spearman ρ = 0.46, *p* < 0.01), together with the association between EDSS and thalamic/hippocampal atrophy in regression analyses, is consistent with a clinically meaningful cross-sectional association between structural damage and functional disability. The cross-sectional design precludes causal inference, and longitudinal data will be required to determine whether regional atrophy precedes, parallels or follows disability accumulation at the individual-patient level.
(5) The present findings are predominantly descriptive and hypothesis-generating. Owing to the cross-sectional design, the absence of formal cognitive assessment, the lack of harmonised lesion burden and treatment data, and the modest single-centre sample, this study does not permit inferences regarding the underlying mechanisms or the directionality of the observed structural changes, so the reported regional volumetric measures cannot be used as standalone clinical markers. Prospective longitudinal and multi-centre validation will be required before any clinical translation.
(6) Overall, given the cross-sectional design and the absence of lesion burden, disease phenotype, disease-modifying therapy and inflammatory activity covariates from the regression models, the present findings should be interpreted as predominantly descriptive and hypothesis-generating. They do not allow for inferences regarding the underlying mechanisms or the directionality of the observed structural changes, and the regional volumetric measures reported here cannot be used as standalone clinical markers without prospective longitudinal and multi-centre validation.

## Figures and Tables

**Table 1:** Demographic and clinical characteristics of study participants.

Group	n	Age (Years, Mean ± SD)	Female	Male	Lasting (Years)	EDSS (Mean ± SD)
All MS	40	41.9 ± 11.1	28 (70%)	12 (30%)	9.1 ± 8.4 (0.6–30.0)	3.6 ± 1.7 (1.0–6.0)
MS ≤ 5 years	17	34.9 ± 7.2	10 (59%)	7 (41%)	2.2 ± 1.4 (0.6–5.0)	2.6 ± 1.5 (1.0–5.5)
MS > 5 years	23	47.1 ± 10.7	18 (78%)	5 (22%)	14.3 ± 7.6 (6.0–30.0)	4.3 ± 1.4 (1.5–6.0)
Controls	40	40.8 ± 11.0	28 (70%)	12 (30%)	-	-

## Data Availability

Aggregated statistical outputs are available upon reasonable request to the corresponding author, subject to applicable data protection regulations.
